# Differential regulation of the rainbow trout (*Oncorhynchus mykiss*) MT-A gene by nuclear factor interleukin-6 and activator protein-1

**DOI:** 10.1186/1471-2199-14-28

**Published:** 2013-12-17

**Authors:** Peter Kling, Carina Modig, Huthayfa Mujahed, Hazem Khalaf, Jonas von Hofsten, Per-Erik Olsson

**Affiliations:** 1 Örebro Life Science Center, School of Science and Technology, Örebro University, Örebro SE-701 82, Sweden; 2 Department of Zoology, Göteborg University, Göteborg SE-405 30, Sweden; 3 Department of Molecular Biology, Umeå University, Umeå SE-901 87, Sweden

**Keywords:** Rainbow trout, Metallothionein-A promoter, Nuclear factor interleukin-6, Activator protein-1, Oxidative stress

## Abstract

**Background:**

Previously we have identified a distal region of the rainbow trout (*Oncorhynchus mykiss*) metallothionein-A (rtMT-A) enhancer region, being essential for free radical activation of the rtMT-A gene. The distal promoter region included four activator protein 1 (AP1) cis-acting elements and a single nuclear factor interleukin-6 (NF-IL6) element. In the present study we used the rainbow trout hepatoma (RTH-149) cell line to further examine the involvement of NF-IL6 and AP1 in rtMT-A gene expression following exposure to oxidative stress and tumour promotion.

**Results:**

Using enhancer deletion studies we observed strong paraquat (PQ)-induced rtMT-A activation via NF-IL6 while the AP1 cis-elements showed a weak but significant activation. In contrast to mammals the metal responsive elements were not activated by oxidative stress. Electrophoretic mobility shift assay (EMSA) mutation analysis revealed that the two most proximal AP1 elements, AP1_1,2_, exhibited strong binding to the AP1 consensus sequence, while the more distal AP1 elements, AP1_3,4_ were ineffective. Phorbol-12-myristate-13-acetate (PMA), a known tumor promoter, resulted in a robust induction of rtMT-A via the AP1 elements alone. To determine the conservation of regulatory functions we transfected human Hep G2 cells with the rtMT-A enhancer constructs and were able to demonstrate that the cis-elements were functionally conserved. The importance of NF-IL6 in regulation of teleost MT is supported by the conservation of these elements in MT genes from different teleosts. In addition, PMA and PQ injection of rainbow trout resulted in increased hepatic rtMT-A mRNA levels.

**Conclusions:**

These studies suggest that AP1 primarily is involved in PMA regulation of the rtMT-A gene while NF-IL6 is involved in free radical regulation. Taken together this study demonstrates the functionality of the NF-IL6 and AP-1 elements and suggests an involvement of MT in protection during pathological processes such as inflammation and cancer.

## Background

Reactive oxygen species (ROS) are continuously being generated during oxygen dependent events in living organisms. ROS production is highly correlated to pathological responses both at the cellular and organismal level [1]. These responses include events such as cancer, cell death and aging. At the cellular level the oxidative stress response results in activation and up regulation of several antioxidant enzymes, such as superoxide dismutase (SOD), catalase and glutathione-s-transferase (GST) as well as non-enzymatic antioxidant compounds, such as ascorbic acid, β-carotene and reduced glutathione, GSH [2]. The role of metallothionein (MT) as a free radical scavenger has been well documented in vitro [3], in cell lines [4], and at the organism level [5,6]. Furthermore, it has been shown that MT is induced by ROS inducing agents such as paraquat (PQ), hydrogen peroxide (H_2_O_2_) and glutathione depleting agents such as diethylmaleate [1,4,7]. In addition, Phorbol-12-myristate-13-acetate (PMA) is a potent tumor promoter shown to induce MT expression via activator protein 1 (AP1) cis-acting elements in mammals [8-10].

Sequencing of several teleost MT enhancer regions have revealed the presence of distally located nuclear factor interleukin 6 (NF-IL6) and AP1 elements, inferring that these elements are involved in a conserved function [7,11-13]. The transcription factor NF-IL6 is activated by the cytokine IL-6 in response to NF-κB activation. NF-IL6 is suppressed in normal tissues, but is rapidly and drastically induced by inflammation [14].

The composite transcription factor AP1 was first identified as a factor mediating optimal basal level expression of the human MT2A (hMT2A) enhancer region [15-17]. Sequencing of the Fugu genome allowed determination of gene similarity between human and teleost genomes [18]. It has been shown that there are more AP1 genes in Fugu than in mammals and that they share high homology in the DNA binding and dimerisation domains [19]. The sequence data thus indicate that the functionality of the AP1 proteins may be highly similar in both teleosts and mammals.

MT-I induction by ROS in mouse has shown to be mediated by metal responsive elements (MREs) and antioxidant responsive elements (ARE)/upstream stimulatory factor (USF) cis-acting elements [20]. The USF/ARE composite transcription factor has also been identified in a number of other terrestrial vertebrates including chicken [21]. The USF cis-acting element has been shown to participate in basal level transcription of the mouse MT-I gene [22]. The ARE cis-acting element has also been identified and characterized in enhancer regions from metabolizing enzymes participating in the phase II drug response and is activated by electrophilic xenobiotics and H_2_O_2_[23]. Oxidative stress has been hypothesized to be the main driving force for gene activation via ARE [24,25]. However, it has been indicated that ARE driven gene expression can occur in the absence of oxidative stress through the transcription factor nuclear factor (erythroid-derived 2)-like 2 (Nrf-2) [26]. The AP1 cis-acting element share high homology to the ARE but is not a component of the protein complex that binds the USF /ARE site on the mouse MT I enhancer region [27]. However, AP1 has been shown to bind to ARE in the NADP (H): quinone oxidoreductase hNQO1 gene enhancer resulting in activation of this gene [28,29].

Functional analysis of the rtMT-A promoter has indicated that free radicals regulate the rtMT-A gene via a region containing multiple copies of AP1 elements and one NF-IL6 element [7]. In the rtMT-A gene there is a distal region of the promoter that contain AP1 elements with a potential to regulate MT gene expression. Upstream of the AP1 elements there is one distinct NF-IL6 element that has not been previously analyzed for its involvement in MT regulation. In studies of teleosts there is no clear correlation between the presence of AP1 elements and free radical responsiveness of the MT genes [30,31]. However, a study on the interaction between MT and free radicals indicate that rtMT-A and sea mussel (*Mytilus galloprovincialis*) MT10 has a higher ROS scavenging capacity than rabbit MT [32]. Furthermore, in an open sea study on cod, a strong correlation was observed between hepatic MT levels and the total ROS scavenging capacity of the liver [33]. Thus, while AP1 has not been clearly correlated with MT induction in teleosts it remains that one of the functions of MT is ROS regulation. To further explore the understanding of MT regulation in teleosts by ROS and tumor promoting agents we have analyzed the relative contribution of AP1 and NF-IL6 elements in the rtMT-A enhancer region. In order to study the conservation of the identified cis-elements we tested the regulatory potential using both homologous as well as heterologous systems. Functional analysis of the rtMT-A promoter suggest that the NF-IL-6 element is instrumental to MT induction by oxidative stress while the AP-1 elements exhibited a strong activation in response to tumor promoting agents such as PMA. The AP-1 elements appeared only to a minor extent contribute to free radical inducibility of the rtMT-A gene. Moreover, hepatic expression of rtMT-A mRNA was substantially increased in response to both oxidative stress and tumor promotion, suggesting that MT may be involved in the protection against pathological processes such as inflammation and cancer.

## Results

### Basal level expression of the rtMT-A promoter

**Figure 1:**
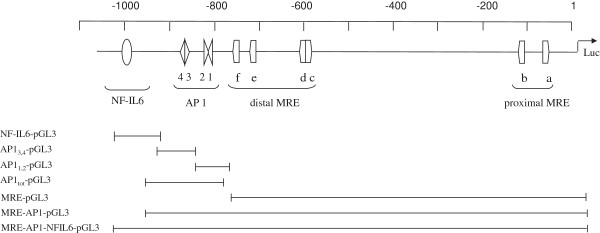
**Cis-acting elements of the rtMT-A promoter.** The location and orientation of the NF-IL6, AP1 and MRE elements on the rtMT-A enhancer region is shown. There are 6 MREs (MRE), 4 AP1 (AP1_tot_) and 1 NF-IL6 (NF-IL6) element located in the first 1100 bp upstream of the transcriptional start site in the rtMT-A gene. The constructs shown are -1042/-917 that contain the NF-IL6 element alone; -939/-812 that contain AP1_3,4_; -834/-774 that contain AP1_1,2_; -939/-774 (AP1_tot_); -793/+23 (MRE); -939/+23 (MRE-AP1); -1042/+23 (MRE-AP1-NFIL6). The TATA box found in rtMT-A is included in the reverse primers of the distally located constructs. The constructs were cloned into the pGL3-basic vector.

**Figure 2:**
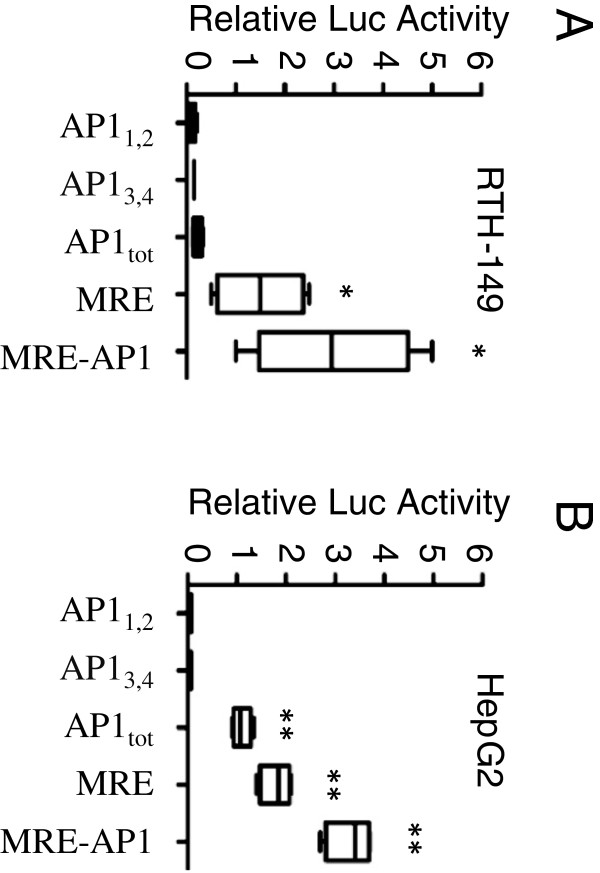
**Basal level expression of rtMT-A cis-acting elements.** Basal level luciferase activity in RTH-149 **(A)** and Hep G2 **(B)** cells transfected with the indicated rtMT-A promoter constructs. Luciferase activity is given as relative luminescence. All activities were normalized using either the dual luciferase system or β-galactosidase activity. Luciferase activities were analyzed 24 hours post transfection. Results are presented as mean ± SE (n = 4). All other activities were adjusted accordingly. Statistically significant differences from control levels are indicated by *(p < 0.05); ** (p < 0.01).

Transfection of the indicated deletion constructs (Figure 1) in both homologous (RTH-149) and heterologous (Hep G2) systems show that the full-length rtMT-A promoter is required for maximal basal level expression (Figure 2). While single pairs (AP1_1,2_ and AP1_3,4_) of AP1 elements did not initiate transcription, the region containing the complete cluster of AP1 elements (AP1_tot_) enhanced basal level activity 2-fold and 20-fold in the RTH-149 and Hep G2 cell lines respectively. The MRE construct (-793, complete set of 6 MREs) was observed to be important for basal level activity of the rtMT-A promoter in both cell lines. Transfection with the MRE-AP1 (-939, complete set of 6 MREs and 4 AP1 elements) construct resulted in further enhancement of basal level expression.

### rtMT-A activity following PQ and H_2O2_ exposure

**Figure 3:**
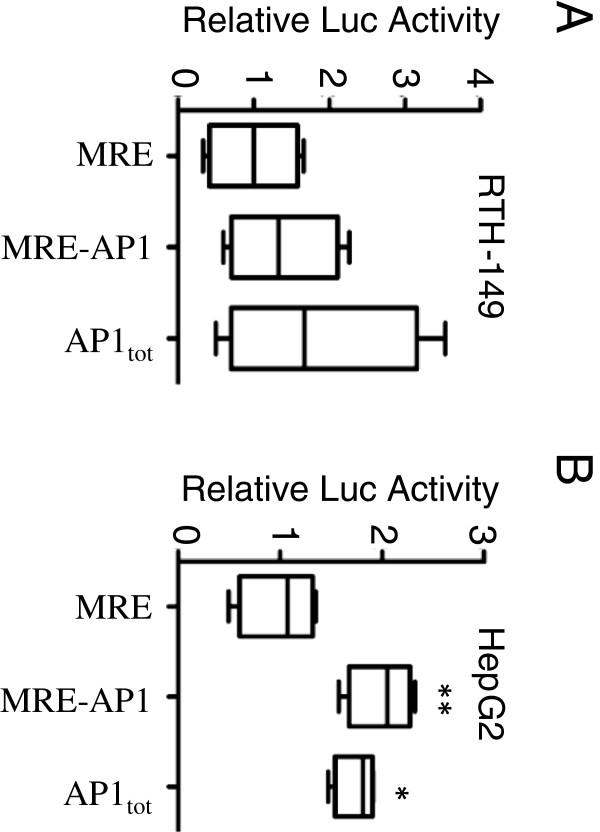
**Activation of rtMT-A cis-acting elements following exposure to oxidative stress.** Luciferase activity in RTH-149 **(A)** and Hep G2 **(B)** cells transfected with the indicated rtMT-A enhancer vectors. Cells were exposed to 10 μM PQ (RTH-149) or 100 μM H_2_O_2_ (Hep G2). Activities are given as fold induction. All activities were normalized using either the dual luciferase system or β-galactosidase activity. Luciferase activities were analyzed 24 hours post transfection. Results are presented as mean ± SE (n = 4). Statistically significant differences from control levels are indicated by *(p < 0.05); ** (p < 0.01).

RTH-149 and Hep G2 cells were transfected with the MRE-pGL3 vector (-793), the MRE-AP1-pGL3 vector (-939), and the AP1-pGL3 vector (AP1_tot_). Treatment of transiently transfected RTH-149 cells with 10 μM PQ (Figure 3A) or 100 μM H_2_O_2_ (data not shown) did not result in a significant increase in luciferase activity. However, transfection of Hep G2 cells followed by 100 μM H_2_O_2_ exposure resulted in ~1.5-fold induction with both constructs containing the 4 AP1 elements (Figure 3B). Transfection with the MRE-pGL3 vector did not result in up-regulation of the luciferase expression. These data suggest that the AP1 elements of the rtMT-A promoter alone confer free radical inducibility.

### Mutation analysis and EMSA of rtMT-A AP1 elements

**Figure 4:**
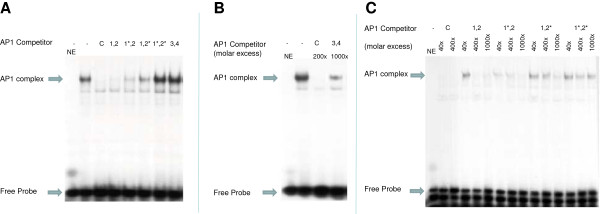
**AP1 binding affinity to AP1 consensus sequence.** Electrophoretic mobility shift assay (EMSA) on HeLa nuclear extract (10 μg). The AP1 consensus oligonucleotide was labelled with γ-32P ATP. Synthetic oligonucleotides, corresponding to the rtMT-A promoter were used as competitors. The constructs used as competitors were: Consensus AP1 **(C)**; AP1_1,2_ (1,2); AP1_1*,2_ (1*,2); AP1_1,2*_ (1,2*); AP1_1*,2*_ (1*,2*) and AP1_3,4_ (3,4). Mutated AP1 elements are indicated by an asterisk (*). NE, no extract. The location of the AP1 protein complex and free unbound probe is indicated by arrows. **(A)** Competitor oligonucleotides were added in 400 x molar excess. **(B)** The AP1 consensus and AP1_3,4_ were added in 200 x and 10,000 x molar excess respectively. **(C)** Dose–response analysis of AP1 oligonucleotides. Synthetic oligonucleotides, corresponding to the AP1 elements in the rtMT-A promoter were used as competitors. Competitor oligonucleotides were added in 40 x, 400 x and 1000 x molar excess.

EMSAs were performed to identify relative binding affinity of the four AP1 elements (the sequences were tested pairwise) of the rtMT-A promoter to the AP1 consensus sequence. A set of normal and mutated AP1 oligonucleotides were used in EMSA competition assays (native and mutated (*) AP1 sequences are presented in Table 1). A strong gel-shift was observed following incubation with labeled AP1 consensus oligonucleotide with HeLa nuclear extracts. This shift was completely abolished by competing with 400-fold excess of either unlabeled AP1 consensus or AP1_1,2_ oligonucleotides (Figure 4A). Mutation of both proximal AP1 elements (AP1_1*,2 *_) abolished AP1 binding while separate mutation of the individual elements (AP1_1*,2_ and AP1_1,2*_) resulted in reduced AP1 binding. Thus, both AP1_1_ and AP1_2_ were observed to be important for interaction with the AP1 protein complex, with AP1_2_ exhibiting strongest affinity. The distally located AP1 elements, AP1_3,4_ showed low binding affinity to the AP1 consensus sequence. However, using a 1000 × molar excess of competitor a slight reduction in the intensity of the shift could be observed (Figure 4B). Dose–response competition of native and mutated AP1_1,2*_ oligonucleotides (40, 400 and 1000 × excess) indicate that a 40 fold excess of consensus AP1 element completely competed away the labeled AP1 element. The AP1_1,2_ oligonucleotide was less effective and requires 400 fold excess for complete competition. The two single mutated sequences (AP1_1*,2_ and AP1_1,2*_), also showed dose-dependent competition although requiring higher concentrations of competitor. Mutation of both the proximally located AP1 elements (AP1_1,2*_) resulted in abolished competition with the AP1 consensus oligonucleotide. The present functional analysis of the rtMT-A AP1 elements demonstrates that the proximally located AP1_1_ and AP1_2_ elements show the highest competition with the AP1consensus sequence.

**Table 1:** Oligonucleotides used for EMSA competition binding assays

**Primer**	**Sequence**
AP1-1,2	TGG TAT GAC ACA GCT C AA TTA CTC AAG CAG
AP1-1*,2	TGG TAT GAC ACA GCT C AA TTA ATT AAG CAG
AP1-1,2*	TGG TAT AAT ACA GCT CAA TTA CTC AAG CAG
AP1-1*,2*	TGG TAT AAT ACA GCT CAA TTA ATT AAG CAG
AP1-3,4	CTG GTA CTG TCA GTG ACT ATT T

The different AP1_1,2_ and AP1_3,4_ oligonucleotides corresponds to position (-814 to -785) and (-867 to -836) of the rtMT-A promoter respectively. The asterisk (*) indicates a mutated cis-element.

### *In vitro* inhibition of H_2_O_2_ induced rtMT-A gene activity

**Figure 5:**
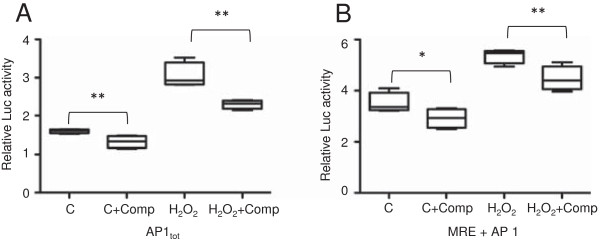
***In vitro *competition assay.** Hep G2 cells were transfected with the 4AP1-pGL3 vector (AP1_tot_) **(A)** or 6MRE-4AP1-pGL3 vector (MRE-AP1) **(B)** alone or together with 50 x molar excess of the AP1_1-2_ and AP1_3-4_ oligonucleotides. Control groups incubated without exposure (Control vs. Control-Comp), while experimental cells were exposed to 200 μM H_2_O_2_ (H_2_O_2_ vs. H_2_O_2_ Comp). Results are presented as mean ± SE (n = 4). Statistically significant differences between groups are indicated by *(p < 0.05); **(p < 0.01).

Transfection of Hep G2 cells with AP1-pGL3 and MRE-AP1-pGL3 vectors resulted in a 2-fold, increase in luciferase activity following H_2_O_2_ exposure. The H_2_O_2_ induced luciferase activity in cells transfected with AP1 containing constructs was significantly reduced when co-incubated with synthetic double stranded AP1_1,2_ and AP1_3,4_ oligonucleotides, suggesting that the H_2_O_2_ induced gene activity was AP1-specific (Figure 5). The observed reduction in luciferase activity following oligonucleotide competition was strongest when transfecting with the AP1-pGL3 vector. Moreover, as in the previous experiments, there was no up regulation following transfection of the MRE-pGL3 vector alone (data not shown). A decrease in luciferase activity was also observed in control cells co-incubated with competitor. However this decrease was small compared to the observed decrease in cells exposed to H_2_O_2_.

### Activity of AP1 and NF-IL6 elements in response to PQ and PMA

**Figure 6:**
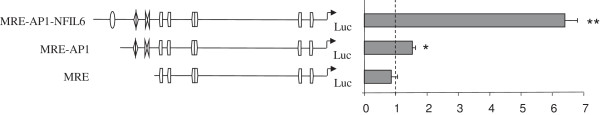
**Activation of rtMT-A deletion constructs following exposure to PQ.** RTH-149 cells were transfected with 0.5 ug of MRE-AP1-NFIL6-pGL3, MRE-AP1-pGL3 and MRE-pGL3 vectors and 0.3 μg of pRL-CMV vector. Following transfection cells were exposed to 10 μM PQ. The activities are given as fold induction. All activities were normalized to the expression level of the pGL3-basic vector. Luciferase activities were analyzed 24 hours post transfection. Results are presented as mean ± SE (n = 4). Statistically significant differences from control levels are indicated by *(p < 0.05); **(p < 0.01).

**Figure 7:**
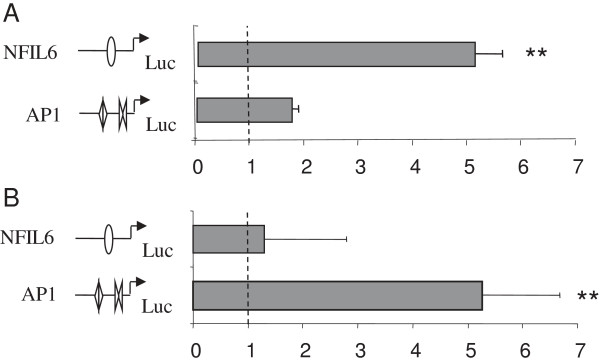
**Activation of rtMT-A deletion constructs following exposure to PQ and PMA.** RTH-149 cells were transfected with 0.5 μg of either the NFIL6-pGL3 or the AP1-pGL3 (AP_tot_) vectors and 0.3 μg of pRL-CMV vector (Renilla Luciferase control reporter vector). Following transfection cells were exposed to 10 μM PQ **(A)** or to 162 nM PMA **(B)**. The activities are given as fold induction. All activities were normalized to the expression level of the pGL3-basic vector. Luciferase activities were analyzed 24 hours post transfection. Results are presented as mean ± SE (n = 4). Statistically significant differences from control levels are indicated by **(p < 0.01).

Exposure of RTH-149 cells to 10 μM PQ resulted in a 6-fold increase in gene activity following transfection with the MRE-AP1-NF-IL6-pGL3 vector containing the distally located NF-IL-6 element (Figure 6). Transfection with the MRE-AP1-pGL3 vector resulted in a modest increase in luciferase activity while the MRE-pGL3 vector did not elicit an increased luciferase activity. The response to 10 μM PQ was similar following transfection with the NF-IL6-pGL3 vector (Figure 7A), indicating that the NF-IL6 enhancer mediated the observed PQ inducibility. In contrast, transfection with the AP1-pGL3 vector and exposure to 10 μM PQ did not result in an increased luciferase response, suggesting that the AP1 elements are not primarily involved in the PQ response. On the other hand, exposure to 162 nM PMA resulted in a robust induction (5-fold) of luciferase activity following transfection with the AP1-pGL3 vector. The isolated NF-IL-6 element was unresponsive to PMA (Figure 7B). Thus, both the AP1 and the NF-IL6 elements were functional in rainbow trout cells.

### *In vivo* induction of MT-A mRNA by PQ and PMA

**Figure 8:**
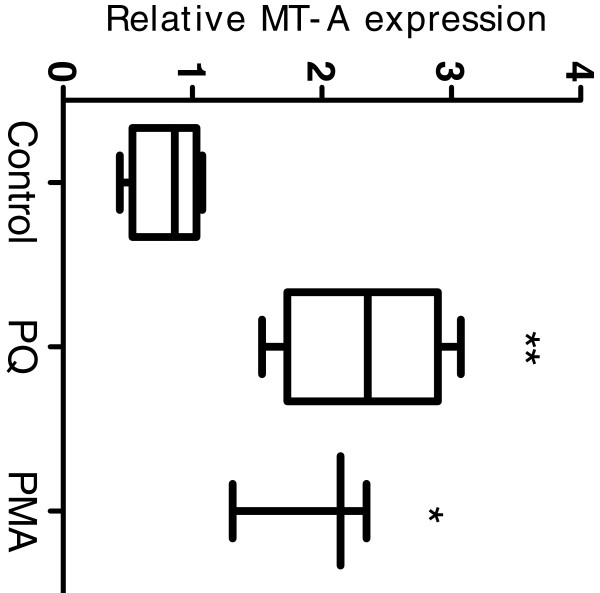
**Hepatic MT mRNA expression following injection with PQ or PMA.** Real-time quantitative PCR was used to determine the relative gene expression levels of the rtMT-A gene. Control fish were injected with 1% DMSO, while experimental fish were injected with 10 μM PQ or PMA and exposed for 24 hours. Results are presented as mean ± SE (n = 5). Expression levels were normalized against EF1α using ∆∆CT method. Statistically significant differences from control levels are indicated by *(p < 0.05); ** (p < 0.01).

Rainbow trout were injected with 10 mg/kg PQ and 10.3 μg/kg PMA in order to determine the effect on endogenous hepatic MT-A gene expression. A 2.5- and a 2-fold induction of hepatic MT-A mRNA was observed following exposure to PQ and PMA respectively (Figure 8), confirming that both inducers result in up regulation of rtMT-A mRNA in vivo.

## Discussion

As a consequence of organism utilization of oxygen, deleterious reactive oxygen species are produced. These oxygen species may lead to lipid peroxidation, DNA strand breaks and cell death. However, several antioxidant systems, such as GSH, SOD, catalase and MT have evolved to protect the organism from oxidative stress. A wide variety of stresses, ranging from physical injury to oxidative stress, induce MT in animals [27,34]. Although numerous studies have been performed to confirm MTs antioxidative function, few have focused on the link between regulation of MT and a role during oxidative stress. In the present study we aimed to characterize the regulatory role of the rtMT-A enhancer region in response to oxidative stress and tumor promotion. Previous identification and functional analysis of distal elements on the rtMT-A promoter have revealed that a cluster including 4 AP1 elements and a single NF-IL-6 element may be involved in free radical inducibility [7]. While free radical regulation of mammalian MT genes seem to be mediated via USF/ARE and MRE cis-acting elements [20], teleost MT genes may be regulated via conserved clusters of cis-acting elements sharing high homology to the NF-IL6 and the AP1 consensus core sequences [7,11,12]. Functional analysis, from the present study, of the rtMT-A promoter suggest that the NF-IL-6 element is instrumental to MT induction by oxidative stress (PQ), and the AP-1 elements may to a minor but significant extent contribute to free radical inducibility.

The observed absence of ROS induced MRE activation in the present study demonstrates that MT regulation in rainbow trout differ from that in mammals where the MRE binding transcription factor MTF-1 is activated following oxidative stress [35]. Since the MRE elements identified on the rtMT-A promoter were not observed to contribute to the oxidative response this suggests that teleost MTF-1 is not activated by oxidative stress. Characterization of MTF-1, from zebrafish and rainbow trout has revealed a high conservation with regard to binding specificity and properties [36]. However, this study points out that there might be different mechanisms that regulate MT gene expression during oxidative stress in different species.

While USF/ARE and MTF-1 mediate oxidative MT expression in mammals, the AP1 cis-acting elements identified on the rtMTA promoter, sharing high homology to the ARE core sequence, showed weak activation in response to oxidative stress. The AP1 cis-acting element was originally discovered on the hMTIIa gene mediating optimal basal level expression of MT [37]. Functional analysis of the identified rtMT-A elements strongly indicated that the AP1 elements were required for maximal basal level expression in both fish (RTH-149) and mammalian cell (Hep G2) systems. Further analysis of binding affinity for the AP1 consensus sequence indicate that the proximal AP1 pair exhibited highest binding affinity, while the binding activity of the distal AP1 elements was at the border of detection limit. In addition, mutational analysis indicated that AP1_2_ showed highest binding of the proximally located pair. Hence, functional analysis of the identified AP1 elements suggests that AP1_1,2_ is functional with respect to both AP1 protein complex interaction and transactivation. However, there have been conflicting reports on the involvement of AP1 in the free radical regulation of MT in teleosts [30,31]. While a deletion construct containing MRE and AP1 responded to ROS in common carp [30], the zebrafish MT gene promoter that contains both AP1 and MRE elements did not respond to ROS [30,31]. In the present study co-transfection of Hep G2 cells with rtMT-A AP1 containing constructs with rtMT-A AP1 oligonucleotides abolished H_2_O_2_ induced promoter activity. These data suggest that the identified AP1 elements on the rtMT-A promoter in rainbow trout specifically mediate free radical MT inducibility. The primary role of the AP1 protein complex is as a modulator of cell proliferation and differentiation. It has been indicated that there is a link between proliferating human hepatic cells and high expression of MT protein [38]. Exposure of RTH-149 cells to the tumor promoter PMA resulted in a strong activation of AP1 cis-elements in the present study. In vivo injection of rainbow trout with PMA strongly induced hepatic rtMT-A mRNA levels, confirming the functionality of the AP1 elements on the rtMT-A promoter. These data strengthen the view of MT as a modulator of cell proliferation and differentiation. In the teleost CHSE-214 cell line MT becomes progressively methylated during prolonged subculturing, coinciding with a decrease in MT expression and reduced cell proliferation [39]. It has been suggested that antioxidants such as ascorbate, α-tocopherol and β-carotene are inhibitory to differentiation [40]. MT has also been suggested to alter the cellular redox state [41], indicating a role for MT during development and differentiation.

Cellular responses following stress, such as injury, promote a transient activation of NF-IL6 and AP1 protein that both play key roles in the initiation of inflammatory responses. NF-IL6 is rapidly activated in response to cytokines and oxidative stress [42]. This is in support with the present study in the RTH-149 cell line demonstrating that the NF-IL6 element was substantially activated in response to PQ but not to PMA. NF-IL6 sites are also present in the MT enhancer of other teleosts such as the crucian carp [12] suggesting a conserved function of MT in response to inflammation where NF-IL6 may mediate ROS inducibility. Studies have demonstrated that NF-IL6 activity can be modulated following protein-protein interaction with the AP1 protein complex [43] and NF-κB [14]. The observed minor increase in AP1 element activity following PQ exposure may enhance MT expression to protect from oxidative stress in fish. Rainbow trout cells respond to ROS exposure by induction of MT, which result in cellular protection from ROS toxicity [3,4,6]. Furthermore, it has been shown in a recent study on cod that there is a close correlation between the hepatic levels of ROS and MT [33]. IL6 and CXCL8 are two of the first inflammatory mediators expressed and contain cis-acting sites for both AP1 and NF-IL6, which indicate their crucial role in acute phase responses [44,45]. Kanekiyo and colleagues [46] demonstrated that MT gene activation by zinc regulates macrophage colony stimulating factor (M-CSF), which in turn recruit and stimulate cytokine production by monocytes. Mice with genetic deletions in the MT proteins have a significant reduction in inflammatory mediators, including TNFα, IL6 and IL1α, compared to wild type mice [47]. Furthermore, growth hormone (GH) was found to induce the expression of rtMT-A) [48]. GH treatment results in phosphorylation of NF-IL6 and increases its transcriptional activity. This suggests that GH may be able to modulate MT regulation through NF-IL6 signaling.

## Conclusions

The present study demonstrates that the NF-IL6 and AP1 cis-acting elements of the rtMT-A promoter are functionally active. While NF-IL6 was instrumental for MT induction by oxidative stress, the AP1 elements was primarily and substantially activated in response to tumor promotion. Since NF-IL6 is a key component of initiation of inflammation it appears that MT may regulate this process by free radical scavenging. These data strengthens the idea of MT as an important regulator of proliferation and differentiation. In addition, there appears to be a complex interplay between NF-IL6 and AP1 that needs to be addressed in future studies to understand the link between MT expression, inflammation, development and differentiation.

## Methods

### Construction of luciferase plasmids

PCR was used to construct the desired rtMT-A promoter luciferase plasmids. The previously sequenced 5′flanking region of the rtMT-A enhancer region was used as a template for all PCR reactions. Forward and reverse primers, containing a Kpn I and Hind III site respectively were used to create the desired luciferase constructs (Table 2). The binding sites for the PCR primers used to create the different rtMT-A enhancer region constructs are shown in Figure 1. The PCR products were resolved on agarose gels and purified using the Qiagen PCR purification kit. Purified PCR fragments were subsequently cloned into the Kpn I –Hind III site of the luciferase reporter vector pGL3-basic (Promega, USA). The size of each insert was confirmed by restriction digest using the Kpn I and Hind III restriction enzymes. Large preparations of each construct were then performed using the Qiagen Midi kit. Each plasmid construct was resolved on agarose gels to test the purity.

**Table 2:** Oligonucleotides used to create rtMT-A gene promoter deletion mutants

**Primer**	**Location**	Sequence*
NF-IL6 forward	(-1042 to -1020)	GCG **GGT ACC** TAT GTT CGA TTG GAC TAT GAT TC
AP1 forward 1	(-939 to -917)	GCG **GGT ACC** TGA TAG ACT ATC CTT GTT GTA GG
AP1 forward 2	(-834 to -815)	GCG **GGT ACC** ATA ACA TTG CAC AAT GTT TG
MRE forward	(-793 to -773)	CGG **GGT ACC** TCA AGC AGG AGA TTC TGG AA
NF-IL6 reverse	(917 to -939)	GCG **AAG CTT***TTT ATA* TCG CTA CAA TTA ATT ACA AAC GAC CG
AP1 reverse 1	(-774 to -790)	GCG **AAG CTT***TTT ATA* TCG CCA GAA TCT CCT GCT TG
AP1 reverse 2	(-812 to -829)	GCG **AAG CTT***TTT ATA* TCG CCA CAA ACA TTG TGC AAT G
MRE reverse	(+23 to +5)	GCG **AAG CTT** CAG TGG TGT GTT GTC AGC G

*The restriction enzyme sites are shown in bold (the 3′ primers contain a Hind III site and the 5′ primers contain a Kpn I site) and TATAA box sequences are shown in italics. The MRE contains the natural TATAA box found in rtMT-A.

The fish handling procedures were approved by the Swedish Ethical Committee in Umea (Permit A75-01).

### Cell culture conditions

The rainbow trout hepatoma (RTH-149) cells were propagated at 18°C and the human hepatoblastoma (Hep G2) cells were propagated at 37°C. Both cell lines were grown in minimum essential medium with Earle’s salts (GIBCO, Life Technologies) and supplemented with 10% fetal calf serum (FCS) and 1% L-glutamine in an atmosphere of 5% CO_2_^.^

### Transfection

The cells were co-transfected with 0.5 μg of enhancer coupled pGL3-basic vector or the empty pGL3-basic vector and 0.3 g of pRL-CMV vector (Renilla Luciferase control reporter vector, Promega, USA), in serum free medium using Lipofectamin 2000 (Invitrogen, USA). The cells were transfected under serum free conditions for 15 hours. Hep G2 cells were grown in 250 ml tissue culture bottles to semi confluence and were then seeded in 3 cm-diameter culture dishes, at a density of 15,600 cells/cm^2^, 24 hours prior to transfection. The cells were transfected with 1 μg of enhancer coupled pGL3-basic vectors and co-transfected with 1 μg of the pSV-β-galactosidase plasmid containing the simian virus 40 early promoters. In the competition experiments 50 molar excess of synthetic AP1_1,2_ and AP1_3, 4_ (for description of oligonucleotides see Table 1) was co-transfected with the AP1-pGL3 vector or the MRE-AP1-pGL3 vector. The total time of transfection in the competition experiment was 14 hours. It was observed that longer transfection times resulted in reduced effect of the oligonucleotides and was probably due to oligonucleotide degradation.

### Reporter gene assays and exposure to PQ and PMA

Following transfection cells were re-fed with fresh media and allowed to recover for 2–4 hours. Fresh media alone or media containing 10 μM PQ, 162 nM PMA or H_2_O_2_ (100 and 200 μM) was added to the cells. After 24 hours exposure, the cells were washed with phosphate buffered saline (PBS) and harvested using cell lysis buffer (Promega, USA). The lysed Hep G2 cells were centrifuged and the supernatant stored at -20°C until β-galactosidase (control) and luciferase assays were performed. Dual-Luciferase reporter assay system (Promega, USA) was used in RTH-149 cell experiments, using the Renilla luciferase vector (pRL-CMV) as a control. For luciferase assays cell lysate were mixed with luciferase substrate and the luciferase activity of the construct was immediately measured in a luminometer (Turner Designs). The cells co-transfected with pRL-CMV was thereafter measured for Renilla luciferase activity. For β-galactosidase assays cell lysate and substrate buffer were mixed and incubated for 30 minutes or until a faint yellow color appeared. The absorbance was then measured at 420 nm. The luciferase activities in Hep G2 cells were normalized for β-galactosidase activity and expressed as relative luciferase activity or luciferase arbitrary units. The activity in RTH-149 cells was calculated by the quotient of the construct/Renilla luminescence.

### Electrophoretic mobility shift assay (EMSA)

EMSAs were performed using commercially available HeLa (human) nuclear extracts (Promega, USA). Synthetic normal and mutated AP1 oligonucleotides were synthesized (DNA technology, Denmark). The oligonucleotides that were used are described in Table 1. Complementary strands were denatured for 5 minutes at 80°C and allowed to anneal by slow cooling to room temperature. Both strands were labelled with [-32P] ATP and T4 polynucleotide kinase. EMSA mixtures contained 40,000 cpm (~0.2 ng) and 10 μg of nuclear protein in a final volume of 15 μl of 4% glycerol; 1 mM MgCl_2_; 0.5 mM DTT; 50 mM NaCl; 10 mM Tris–HCl (pH 7.5) with 1 μg of poly (dI-dC) as nonspecific competitor. The binding reaction with labeled AP1 consensus oligonucleotide was incubated for 20 minutes at room temperature. Incubation of the competitor DNA fragment, in molar excess, with nuclear protein and binding buffer for 10 minutes at room temperature, was performed to initiate the completion binding experiments. The labeled probe was then added, and incubation was allowed to proceed for another 20 minutes at room temperature. 1.5 μl of 10 × gel-shift loading buffer was added to each sample. The reaction mixtures were then loaded onto 4% non-denaturing polyacrylamide (37.5:1) gels at 250 V for approximately 2 hours. The gels were subsequently dried and autoradiographed at -70°C, using an intensifying screen.

### In vivo PQ and PMA exposure

Fish (100 g) were kept in aquaria for 1 week prior to experimental start. Fish were injected with either PQ (10 mg/kg) or PMA (10.3 μg/kg). All fish received a total volume of 200 μl/100 g fish containing 1.5% DMSO dissolved in PBS (n = 5) Controls received 1.5% DMSO alone. Following injection the fish were kept for 4 days. Fish were killed with a blow to the head and the livers were removed and frozen in liquid nitrogen and stored at -80°C until analyzed.

### Real time qPCR

Total RNA was isolated from liver samples using NucleoSpin RNAII kit (Macherey-Nagel, Germany) and quantified by Nano-vue (GE Healthcare, USA). cDNA was prepared using qScript cDNA synthesis kit (Quanta Biosciences, USA). The qPCR was performed using KAPA SYBR FAST qPCR kit (Kapabiosystems, USA) according to manufacturer’s recommendations. Obtained Ct values were normalized against elongation factor EF1 alpha (EF1α). Relative gene-expression was determined by using the ΔΔCt method [49]. The following primer sequences was used for EF1α forward (5′GCATCAAGCAGTGGTCGAGTGA′3), EF1α reverse (5′TTGAAAGAGCCCTTGCCCATCTCA′3), rtMT-A forward, (5′ACACCACTGACACCCAGACAAACT′3) and rtMT-A reverse (5′AGCTGGTATCACAAGTCTTGCCCT′3).

### Statistical analysis

Statistical differences was determined using the two-tailed Student *t*-Test. Statistical significance level was determined at the *P < 0.05 and **P < 0.01 level.

## Competing interests

The authors declare that they have no competing interests.

## Authors’ contribution

PEO obtained the fundings. PK, CM and PEO designed the study. PK, CM, HM, HK and JvH carried out the experimental work. All authors drafted, revised and approved the final manuscript.

## References

[B1] Halliwell B Gutteridge JM Free radicals and antioxidant protection: mechanisms and significance in toxicology and disease Hum Toxicol 1988 7 7 13 10.1177/096032718800700102 3278973

[B2] Jones DP Radical-free biology of oxidative stress Am J Physiol Cell Physiol 2008 295 C849 C868 10.1152/ajpcell.00283.2008 18684987 PMC2575825

[B3] Thornalley PJ Vasak M Possible role for metallothionein in protection against radiation-induced oxidative stress. Kinetics and mechanism of its reaction with superoxide and hydroxyl radicals Biochim Biophys Acta 1985 827 36 44 10.1016/0167-4838(85)90098-6 2981555

[B4] Kling PG Olsson P Involvement of differential metallothionein expression in free radical sensitivity of RTG-2 and CHSE-214 cells Free Radic Biol Med 2000 28 1628 1637 10.1016/S0891-5849(00)00277-X 10938459

[B5] Liu J Liu Y Hartley D Klaassen CD Shehin-Johnson SE Lucas A Cohen SD Metallothionein-I/II knockout mice are sensitive to acetaminophen-induced hepatotoxicity J Pharmacol Exp Ther 1999 289 580 586 10087053

[B6] Zheng H Liu J Liu Y Klaassen CD Hepatocytes from metallothionein-I and II knock-out mice are sensitive to cadmium- and tert-butylhydroperoxide-induced cytotoxicity Toxicol Lett 1996 87 139 145 10.1016/0378-4274(96)03770-8 8914622

[B7] Olsson PE Kling P Erkell LJ Kille P Structural and functional analysis of the rainbow trout (*Oncorhyncus mykiss*) metallothionein-A gene Eur J Biochem 1995 230 344 349 10.1111/j.1432-1033.1995.tb20569.x 7601121

[B8] Ebinu JO Stang SL Teixeira C Bottorff DA Hooton J Blumberg PM Barry M Bleakley RC Ostergaard HL Stone JC Ras GRP links T-cell receptor signaling to Ras Blood 2000 95 3199 3203 10807788

[B9] Angel P Poting A Mallick U Rahmsdorf HJ Schorpp M Herrlich P Induction of metallothionein and other mRNA species by carcinogens and tumor promoters in primary human skin fibroblasts Mol Cell Biol 1986 6 1760 1766 3785178 10.1128/mcb.6.5.1760-1766.1986 PMC367704

[B10] Imbra RJ Karin M Metallothionein gene expression is regulated by serum factors and activators of protein kinase C Mol Cell Biol 1987 7 1358 1363 3600629 10.1128/mcb.7.4.1358-1363.1987 PMC365221

[B11] He P Xu M Ren H Cloning and functional characterization of 5′-upstream region of metallothionein-I gene from crucian carp (*Carassius cuvieri*) Int J Biochem Cell Biol 2007 39 832 841 10.1016/j.biocel.2007.01.008 17337234

[B12] Ren H Xu M He P Muto N Itoh N Tanaka K Xing J Chu M Cloning of crucian carp (*Carassius cuvieri*) metallothionein-II gene and characterization of its gene promoter region Biochem Biophys Res Commun 2006 342 1297 1304 10.1016/j.bbrc.2006.02.082 16516146

[B13] Samson SL Paramchuk WJ Gedamu L The rainbow trout metallothionein-B gene promoter: contributions of distal promoter elements to metal and oxidant regulation Biochim Biophys Acta 2001 1517 202 211 10.1016/S0167-4781(00)00273-6 11342100

[B14] Matsusaka T Fujikawa K Nishio Y Mukaida N Matsushima K Kishimoto T Akira S Transcription factors NF-IL6 and NF-kappa B synergistically activate transcription of the inflammatory cytokines, interleukin 6 and interleukin 8 Proc Natl Acad Sci USA 1993 90 10193 10197 10.1073/pnas.90.21.10193 8234276 PMC47740

[B15] Haslinger A Karin M Upstream promoter element of the human metallothionein-IIA gene can act like an enhancer element Proc Natl Acad Sci U S A 1985 82 8572 8576 10.1073/pnas.82.24.8572 3866241 PMC390959

[B16] Karin M Haslinger A Heguy A Dietlin T Imbra R Transcriptional control mechanisms which regulate the expression of human metallothionein genes Experientia Suppl 1987 52 401 405 2959529 10.1007/978-3-0348-6784-9_38

[B17] Scholer H Haslinger A Heguy A Holtgreve H Karin M In vivo competition between a metallothionein regulatory element and the SV40 enhancer Science 1986 232 76 80 10.1126/science.3006253 3006253

[B18] Aparicio S Chapman J Stupka E Putnam N Chia JM Dehal P Christoffels A Rash S Hoon S Smit A Whole-genome shotgun assembly and analysis of the genome of *Fugu rubripes* Science 2002 297 1301 1310 10.1126/science.1072104 12142439

[B19] Cottage AJ Edwards YJ Elgar G AP1 genes in Fugu indicate a divergent transcriptional control to that of mammals Mamm Genome 2003 14 514 525 10.1007/s00335-002-3067-5 12925884

[B20] Dalton T Palmiter RD Andrews GK Transcriptional induction of the mouse metallothionein-I gene in hydrogen peroxide-treated Hepa cells involves a composite major late transcription factor/antioxidant response element and metal response promoter elements Nucleic Acids Res 1994 22 5016 5023 10.1093/nar/22.23.5016 7800494 PMC523772

[B21] Fernando LP Andrews GK Cloning and expression of an avian metallothionein-encoding gene Gene 1994 81 177 183 2806910 10.1016/0378-1119(89)90349-1

[B22] Gregor PD Sawadogo M Roeder RG The adenovirus major late transcription factor USF is a member of the helix-loop-helix group of regulatory proteins and binds to DNA as a dimer Genes Dev 1990 4 1730 1740 10.1101/gad.4.10.1730 2249772

[B23] Jaiswal AK Antioxidant response element Biochem Pharmacol 1994 48 439 444 10.1016/0006-2952(94)90272-0 8068030

[B24] Ishii T Itoh K Takahashi S Sato H Yanagawa T Katoh Y Bannai S Yamamoto M Transcription factor Nrf2 coordinately regulates a group of oxidative stress-inducible genes in macrophages J Biol Chem 2000 275 16023 16029 10.1074/jbc.275.21.16023 10821856

[B25] Rushmore TH Morton MR Pickett CB The antioxidant responsive element. Activation by oxidative stress and identification of the DNA consensus sequence required for functional activity J Biol Chem 1991 266 11632 11639 1646813

[B26] Lee JM Moehlenkamp JD Hanson JM Johnson JA Nrf2-dependent activation of the antioxidant responsive element by tert-butylhydroquinone is independent of oxidative stress in IMR-32 human neuroblastoma cells Biochem Biophys Res Commun 2001 280 286 292 10.1006/bbrc.2000.4106 11162512

[B27] Andrews GK Regulation of metallothionein gene expression by oxidative stress and metal ions Biochem Pharmacol 2000 59 95 104 10.1016/S0006-2952(99)00301-9 10605938

[B28] Venugopal R Jaiswal AK Nrf2 and Nrf1 in association with Jun proteins regulate antioxidant response element-mediated expression and coordinated induction of genes encoding detoxifying enzymes Oncogene 1998 17 3145 3156 10.1038/sj.onc.1202237 9872330

[B29] Venugopal R Jaiswal AK Nrf1 and Nrf2 positively and c-Fos and Fra1 negatively regulate the human antioxidant response element-mediated expression of NAD(P)H:quinone oxidoreductas1 gene Proc Natl Acad Sci USA 1996 93 14960 14965 10.1073/pnas.93.25.14960 8962164 PMC26245

[B30] Chan PC Shiu CK Wong FW Wong JK Lam KL Chan KM Common carp metallothionein-1 gene: cDNA cloning, gene structure and expression studies Biochim Biophys Acta 2004 1676 162 171 10.1016/j.bbaexp.2003.11.009 14746911

[B31] Yan CH Chan KM Cloning of zebrafish metallothionein gene and characterization of its gene promoter region in HepG2 cell line Biochim Biophys Acta 2004 1679 47 58 10.1016/j.bbaexp.2004.04.004 15245916

[B32] Buico A Cassino C Dondero F Vergani L Osella D Radical scavenging abilities of fish MT-A and mussel MT-10 metallothionein isoforms: an ESR study J Inorg Biochem 2008 102 921 927 10.1016/j.jinorgbio.2007.12.012 18243326

[B33] Chesman BS O'Hara S Burt GR Langston WJ Hepatic metallothionein and total oxyradical scavenging capacity in Atlantic cod, *Gadus morhua* caged in open sea contamination gradients Aquat Toxicol 2007 84 310 320 10.1016/j.aquatox.2007.06.008 17659788

[B34] Oh SH Deagen JT Whanger PD Weswig PH Biological function of metallothionein. V. Its induction in rats by various stresses Am J Physiol 1978 234 E282 E285 629343 10.1152/ajpendo.1978.234.3.E282

[B35] Dalton TP Li Q Bittel D Liang L Andrews GK Oxidative stress activates metal-responsive transcription factor-1 binding activity. Occupancy in vivo of metal response elements in the metallothionein-I gene promoter J Biol Chem 1996 271 26233 26241 10.1074/jbc.271.42.26233 8824273

[B36] Dalton TP Solis WA Nebert DW Carvan MJ 3rd Characterization of the MTF-1 transcription factor from zebrafish and trout cells Comp Biochem Physiol B 2000 126 325 335 10.1016/S0305-0491(00)00182-6 11007174

[B37] Lee W Haslinger A Karin M Tijan R Activation of transcription by two factors that bind promoter and enhancer sequences of the human metallothionein gene and SV40 Nature 1987 325 368 372 10.1038/325368a0 3027570

[B38] Studer R Vogt CP Cavigelli M Hunziker PE Kägi JH Metallothionein accretion in human hepatic cells is linked to cellular proliferation Biochem J 1997 328 63 67 9359834 10.1042/bj3280063 PMC1218887

[B39] Kling P Metallothionein regulation and function in teleosts during metal- and free radical exposure 2001 Umea University, Umea, Sweden: Doctoral thesis

[B40] Allen RG Venkatraj VS Oxidants and antioxidants in development and differentiation J Nutr 1992 122 631 635 1542023 10.1093/jn/122.suppl_3.631

[B41] Ye B Maret W Vallee BL Zinc metallothionein imported into liver mitochondria modulates respiration Proc Natl Acad Sci USA 2001 98 2317 2322 10.1073/pnas.041619198 11226237 PMC30136

[B42] Klampfer L Lee TH Hsu W Vilcek J Chen-Kiang S NF-IL6 and AP-1 cooperatively modulate the activation of the TSG-6 gene by tumor necrosis factor alpha and interleukin-1 Mol Cell Biol 1994 14 6561 6569 7935377 10.1128/mcb.14.10.6561-6569.1994 PMC359186

[B43] Hsu W Kerppola TK Chen PL Curran T Chen-Kiang S Fos and Jun repress transcription activation by NF-IL6 through association at the basic zipper region Mol Cell Biol 1994 14 268 276 8264594 10.1128/mcb.14.1.268-276.1994 PMC358376

[B44] Kang SS Woo SS Im J Yang JS Yun CH Ju HR Son CG Moon EY Han SH Human placenta promotes IL-8 expression through activation of JNK/SAPK and transcription factors NF-kappaB and AP-1 in PMA-differentiated THP-1 cells Int Immunopharmacol 2007 7 1488 1495 10.1016/j.intimp.2007.07.011 17761353

[B45] Ondrey FG Dong G Sunwoo J Chen Z Wolf JS Crowl-Bancroft CV Mukaida N Van Waes C Constitutive activation of transcription factors NF-(kappa)B, AP-1, and NF-IL6 in human head and neck squamous cell carcinoma cell lines that express pro-inflammatory and pro-angiogenic cytokines Mol Carcinog 1999 26 119 129 10.1002/(SICI)1098-2744(199910)26:2<119::AID-MC6>3.0.CO;2-N 10506755

[B46] Kanekiyo M Itoh N Kawasaki A Matsuda K Nakanishi T Tanaka K Metallothionein is required for zinc-induced expression of the macrophage colony stimulating factor gene J Cell Biochem 2002 86 145 153 10.1002/jcb.10202 12112025

[B47] Kanekiyo M Itoh N Kawasaki A Matsuyama A Matsuda K Nakanishi T Tanaka K Metallothionein modulates lipopolysaccharide-stimulated tumour necrosis factor expression in mouse peritoneal macrophages Biochem J 2002 361 363 369 10.1042/0264-6021:3610363 11772408 PMC1222316

[B48] Vergani L Lanza C Borghi C Scarabelli L Panfoli I Burlando B Dondero F Viarengo A Gallo G Efects of growth hormone and cadmium on the transcription regulation of two metallothionein isoforms Mol Cell Endocrinol 2007 263 29 37 10.1016/j.mce.2006.08.010 17027146

[B49] Schmittgen TD Livak KJ Analyzing real-time PCR data by the comparative C(T) method Nat Protoc 2008 3 1101 1108 10.1038/nprot.2008.73 18546601

